# The long noncoding RNA MIR210HG promotes tumor metastasis by acting as a ceRNA of miR-1226-3p to regulate mucin-1c expression in invasive breast cancer

**DOI:** 10.18632/aging.102149

**Published:** 2019-08-10

**Authors:** Xiao-yu Li, Li-ye Zhou, Hao Luo, Qi Zhu, Liang Zuo, Gu-yue Liu, Chu Feng, Jun-yong Zhao, Yuan-yuan Zhang, Xue Li

**Affiliations:** 1 Department of General Surgery, Putuo People’s Hospital of Tongji University, Shanghai, China; 2 Department of Medical Oncology, Dana-Farber Cancer Institute, Boston, MA 02215, USA; 3 Department of General Surgery, Shanghai Tenth People’s Hospital of Tongji University, Shanghai, China; 4 Wake Forest Institute for Regenerative Medicine, Wake Forest School of Medicine, Winston-Salem, NC 27101, USA; 5 Department of Pathology, Beijing Chao-Yang Hospital, Capital Medical University, Beijing, China

**Keywords:** MIR210HG, mucin-1c, miR-1226-3p, metastasis, breast cancer

## Abstract

Background: Long noncoding RNAs have been known to be involved in multiple types of malignancies, including invasive breast cancer (IBC). This study aimed to explore the role of long noncoding RNAs in IBC and elucidate the potential molecular mechanisms.

Methods: Using TCGA microarray data analysis, we identified a long noncoding RNA, MIR210HG, highly expressed in IBC. Kaplan-Meier method and the log-rank test were used for survival analysis. The gain-of-function experiments were performed to assess the function of MIR210HG in IBC invasion and migration in both *in vitro* and *in vivo* settings. Bioinformatic analysis as well as luciferase reporter assay, rescue experiments and western blot assay revealed the mode of action of MIR210HG.

Results: The aberrantly enhanced MiR210HG expression predicted poor prognosis and lower survival rate. Knockdown of MiR210HG suppressed IBC cell invasion and metastasis both in *vitro* and in *vivo*. MiR-1226-3p was identified and validated to be the target miRNA of MiR210HG. Furthermore, MiR210HG functions as a competing endogenous RNAs (ceRNA) which sponges miR-1226-3p, therefore upregulates the expression of mucin1 (MUC1-C).

Conclusions: Our study demonstrated that MiR210HG sponges miR-1226-3p to facilitate invasive breast cancer cell invasion and metastasis by regulating mucin-1c and EMT pathway, revealing the oncogenic role of MiR210HG in IBC cells.

## INTRODUCTION

Breast cancer (BC) is the most frequent malignancy in women, with approximately 1.7 million cases diagnosed per year. The severity of this global health issue is underscored by the fact that it is the second leading cause of cancer-related deaths, ~500,000 women annually [1]. Invasive breast cancer (IBC) is a heterogeneous disease that is categorized into several histological subtypes contingent upon the expression status of the hormone receptors: estrogen receptor (ER), progesterone receptor, and HER2. Approximately 75% of IBCs are ER^+^ [2]. BC deaths are primarily caused by metastatic dispersion rather than from the primary tumor [3]. Therefore, early diagnosis of IBC prior to metastasis improves the probability of survival. Although mammograms have made early detection possible, BC is overlooked in 42–50% of cases due to obscured lesions in women with dense breasts [4, 5]. Novel prognostic markers and efficacious therapeutic targets for breast cancer are imperative.

Long non-coding RNAs (lncRNAs), are a diverse class of transcripts greater than 200 nucleotides in length that do not encode proteins. They are transcribed throughout the genome and are involved in physiological and pathological processes [6, 7]. One mechanism by which lncRNAs can function within these processes is by regulating gene expression at the transcriptional and post-transcriptional levels. Accumulating evidence suggests that lncRNAs play a pivotal role in altering cancer cell activities (e.g. proliferation, metastasis, epithelial-mesenchymal transition (EMT), apoptosis, and drug resistance [8–10]).

Recently, the aberrant expression of lncRNAs in invasive breast cancer has been reported. For example, Evans et al. showed the importance of three lncRNAs (HOTAIR, H19 and KCNQ1OT1) in breast tumorigenesis, thereby supporting lncRNA Chromogenic in Situ Hybridization (CISH) as a prospective clinical tool. Notably, CISH permits the identification of spatial expression of lncRNAs within tissue compartments [11]. Richards et al. posit that a subset of lncRNAs have a substantial role in EMT, invasion and metastasis, and therefore are potential therapeutic targets in breast cancers [12]. These studies highlighted the critical roles of lncRNAs in IBC progression, as well as the importance of new biomarker identification in improving the diagnosis of IBC [13, 14].

Recent findings have implicated that lncRNAs can function as competing endogenous RNAs (ceRNAs) for microRNAs (miRNAs) to post-transcriptionally modulate mRNA expression. Yue et al. demonstrated that monitoring MIR210HG expression levels aided in the differentiation of tumor tissue from tumor-adjacent normal tissue with an AUC of 0.8323. This finding suggests the possibility that MIR210HG may be used as a biomarker for glioma diagnosis [15]. Li et al. reported that MIR210HG acts as a sponge for miR-503 to facilitate osteosarcoma cell invasion and metastasis, revealing an oncogenic role for MIR210HG in osteosarcoma [16]. However, its function in IBC progression remains unclear.

The present study investigated the biological function and the underlying mechanism of MIR210HG in IBC. We found that MIR210HG promote IBC progression through inhibition of miR-1226-3p expression. We first detected the expression of MIR210HG and miR-1226-3p in tumor tissues from IBC patients as well as in IBC cell lines. Functional assays showed that MIR210HG promoted proliferation and invasion of MDA-MB-231 and MCF-7 cells. Moreover, MIR210HG promoted the progression of IBC through the miR-1226-3p/MUC1-C axis. Therefore, the study indicated that MIR210HG could act as a promising potential therapeutic target for treating IBC.

## RESULTS

### MIR210HG functions as a potential oncogenic lncRNA and confers a poor prognosis in invasive breast cancer patients

**Figure 1:**
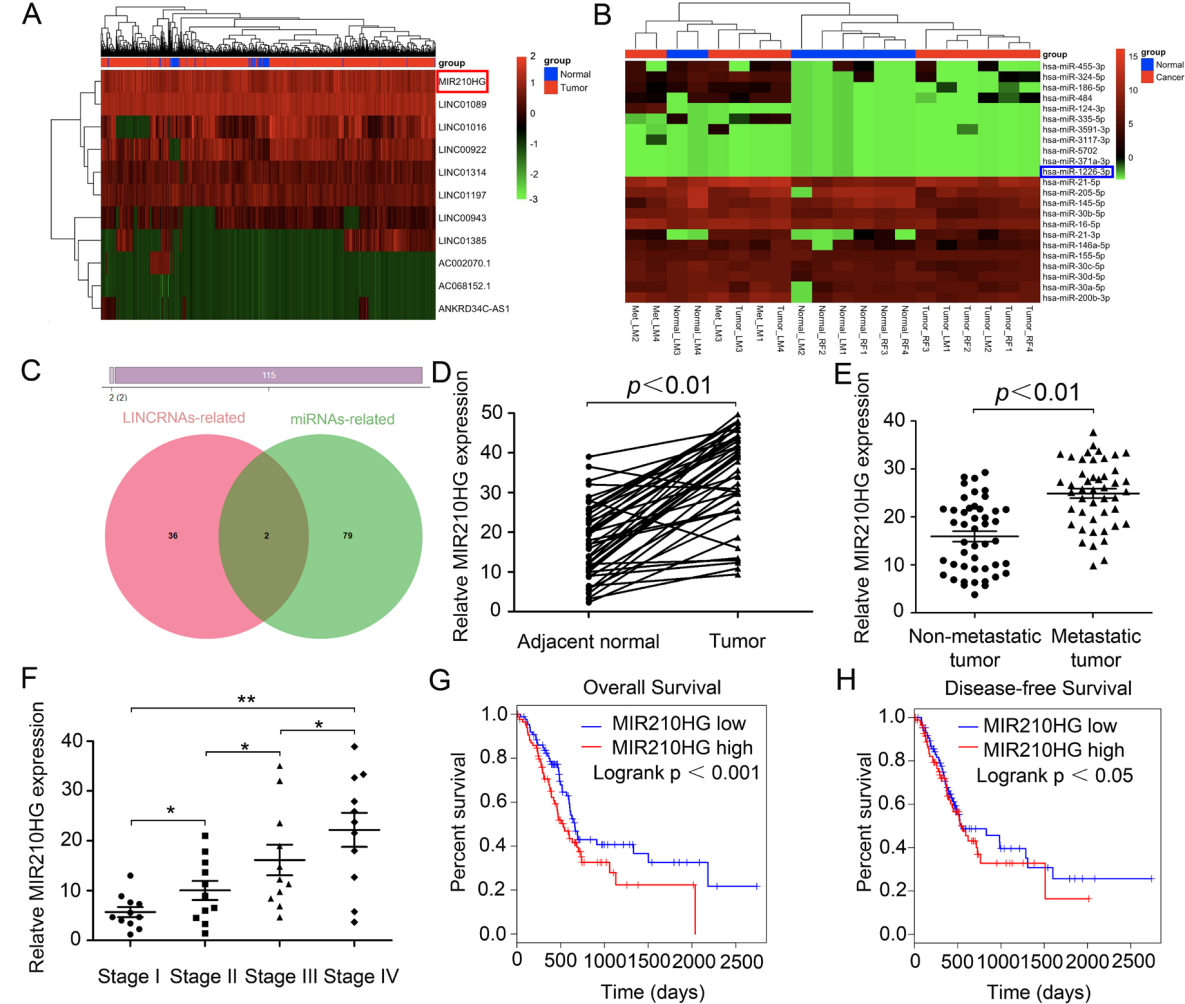
**MIR210HG functions as a potential oncogenic lncRNA and confers a poor prognosis in invasive breast cancer patients.** (**A**) lncRNAs microarray data of invasive breast cancer samples compared with that of normal control tissues are presented in a heat map. Red represents high expression, and green represents low expression. MIR210HG (Ensembl: ENSG00000247095) is listed on the right. (**B**) Heat map of differentially expressed miRNAs from invasive breast cancer miRNA arrays and miRNAs differentially expressed in miRNA arrays. miR-1226-3p (Ensembl: ENSG00000221585) is listed on the right. (**C**) Venn diagrams showing the number of potential non-coding RNAs targeting metastasis-related genes, as predicted by two databases: miRcode and LncBase Predicted. (**D**) Real-time PCR assay shown that MIR210HG is significantly up-regulated in 45 paired fresh IBC tissues. (**E**) Relative expression of MIR210HG in IBC patients with lymph node and distant metastasis. (**F**) Further analysis indicated that TNM stage was positive correlated with MIR210HG. (**G** and **H**) Kaplan-Meier plots of overall-survival (OS) and disease-free survival (DFS) in IBC patients with high and low levels of MIR210HG. *p < 0.05, **p < 0.01.

To investigate differentially expressed noncoding RNAs and their functions in IBC, we initially performed joint analysis of two noncoding RNA arrays (GSE80038 and GSE113851) from GEO datasets by comparing IBC tissues with paired normal tissues. Through GEO array data analysis, we found that a series of lncRNAs including MIR210HG were significantly up-regulated, while miRNAs including miR-1226-3p were significantly down-regulated in IBC compared with normal tissues (Figure 1A and 1B). Next, we selected 115 differentially expressed genes (DEGs) and found that two ncRNAs (MIR210HG and miR-1226-3p) may overlap (Figure 1C). IPA (Ingenuity pathway analysis) results showed that both of them may affect downstream genes involved in tumor metastasis-related pathways. Therefore, we hypothesized that MIR210HG plays an important role in metastasis of IBC, whose function has not been appreciated yet.

To evaluate the oncogenic role of MIR210HG in multiple cancer types, we analyzed the pan-cancer dataset from the TCGA database. We found that the expression of MIR210HG was strikingly increased in BRCA (Breast invasive carcinoma = IBC, TCGA Cell 2015 database), along with HNSC (Head and Neck squamous cell carcinoma, PanCancer Atlas), BLCA (Bladder Urothelial Carcinoma, TCGA, Provisional), CESC (Cervical squamous cell carcinoma and endocervical adenocarcinoma, TCGA, PanCancer Atlas), LUSC (Lung squamous cell carcinoma, TCGA, PanCancer Atlas) and KIRC (Kidney renal clear cell carcinoma, Nature 2016 database) (Supplementary Figure 1), The full-length base sequence and RNA secondary structure information of MIR210HG are displayed in Supplementary Figure 2A–2C, and the methylation level of MIR210HG in different tissues is visually displayed (Supplementary Figure 2D). Additionally, we selected 45 paired IBC patients and determined the MIR210HG expression (using Real-time PCR) in tumor and paired adjacent normal tissues. Consistent with the microarray data, MIR210HG expression was significantly higher in IBC than in the paired non-cancerous normal tissues (Figure 1D, *P* < 0.01). Further analysis indicated that tumor metastasis (including lymphatic metastasis, LM and distance metastasis, DM) and TNM stage were positive correlated with the expression of MIR210HG (Figure 1E and 1F, *P* < 0.01), suggesting that MIR210HG may promote IBC progression, particularly metastasis. In addition, Kaplan-Meier survival analysis suggested that IBC patients with low MIR210HG expression had longer overall survival in TCGA IBC datasets. In this analysis, a total of 1084 breast invasive cancer cases from TCGA with an overall number of 131 events are separated into low (blue) and high (red) expressing group (Figure 1G, Log-rank test, *P* < 0.001). Further, we observed a significant association between disease-free survival and MIR210HG expression level in IBC patients (Figure 1H, *P* < 0.05). Collectively, these data indicate that abnormal MIR210HG expression may be related to IBC patients’ overall survival.

### MIR210HG is up-regulated in breast cancer cell lines MDA-MB-231 and MCF-7, and knockdown MIR210HG inhibits IBC cell proliferation, growth and invasion

**Figure 2:**
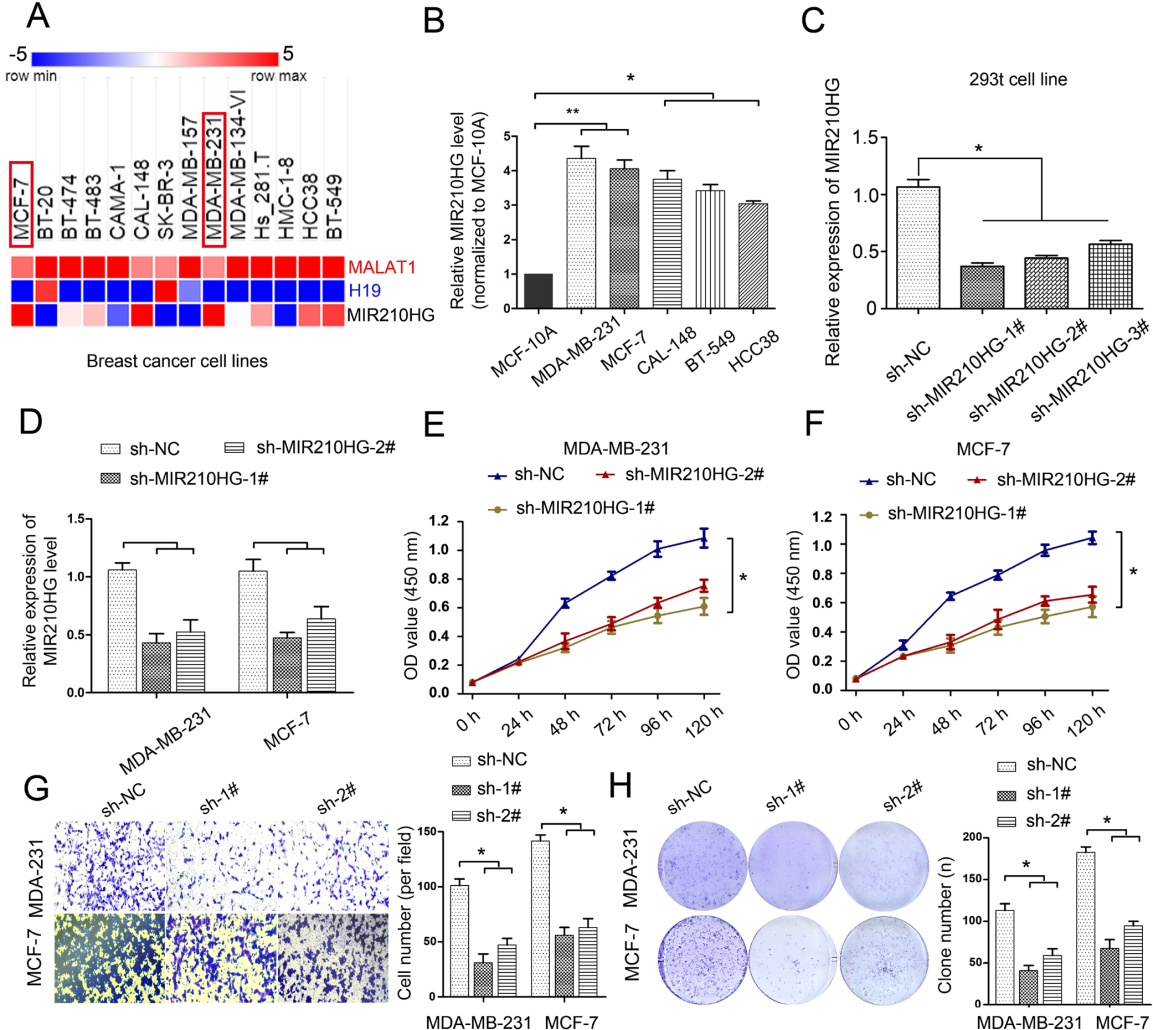
**MIR210HG is up-regulated in breast cancer cell lines MDA-MB-231 and MCF-7, and knockdown MIR210HG inhibits cell proliferation, growth and invasion.** (**A**) MIR210HG data set from the TCGA cell line database (The Atlas of ncRNA in cancer). (**B**) Real-time PCR results confirmed that MIR210HG was increased obviously in MDA-MB-231, MCF-7, 742T, 606T and MDA-MB-134 cell line. (**C**) shRNA-MIR210HG with high interference efficiency in 293t cell. (**D**) MIR210HG knockdown in MDA-MB-231 and MCF-7 transfected with shRNA was determined using qRT-PCR. (**E** and **F**) MTT assay indicated that shRNA-MIR210HG could significantly suppress the proliferation abilities of MDA-MB-231 and MCF-7 cells compared with negative control (sh-NC) group. (**G**) Invasion assay of MDA-MB-231 and MCF-7 cells with shRNA-MIR210HG by transwell assay. (**H**) Colony formation assay revealed that the silencing of MIR210HG greatly reduced the number of colonies of the MDA-MB-231 and MCF-7 cells in comparison with the sh-NC groups. *p < 0.05.

To determine the expression levels of MIR210HG in breast cancer cell lines, we found that 14 IBC cell lines showed various expression levels of MIR210HG in TCGA cell line database. Interestingly, the expression of MIR210HG was higher in IBC cell lines, including MDA-MB-231, MCF-7 and BT-549 (Figure 2A). The expression levels of MAFLAT1 and H19, as 2 well studied long noncoding RNAs, were plotted for comparison with MIR210HG. The expression levels of MIR210HG are independent from that of MALAT1 and H19. Real-time PCR results confirmed that the expression levels of MIR210HG in MDA-MB-231 and MCF-7 were significantly higher than other tested cell line, including CAL-148, BT-459, and HCC38 (Figure 2B, *P* < 0.05). To further define the role of MIR210HG in IBC progression, we selected MDA-MB-231 and MCF-7 for their high expression levels of MIR210HG for subsequent experiments. First, we screened 3 shRNA against MIR210HG with high interference efficiency in 293t cells. Compared with the nontargeting control sh-NC group, all three shRNAs showed significant knockdown of MIR210HG with the highest knockdown efficiency shown by sh-MIR210HG-1# (Figure 2C, *P* < 0.05). Therefore, we used the sh-MIR210HG-1# to inhibit MIR210HG in MDA-MB-231 and MCF-7 cell line (Figure 2D). Furthermore, MTT assay results showed that in both MDA-MB-231 and MCF-7 cells MIR210HG knockdown inhibited the breast cancer cell proliferation (Figure 2E and 2F, *P* < 0.05) and colony formation (Figure 2H, *P* < 0.05). To avoid off-target effect, we repeated the same experiment using another shRNA, sh-MIR210HG-2#. Consistent with previous results, loss of function of MIR210HG resulted low cell proliferation and less colony formation (Figure 2D–2H). The results of transwell assay showed that cell invasion is also remarkably suppressed in sh-MIR210HG-1# transduced cells compared with the control group (Figure 2G, *P* < 0.05). Conversely, when we overexpressed MIR210HG in MDA-MB-231 and MCF-7 cells, we found that the invasiveness of the cells increased significantly, and the expression level of MUC1-C protein also increased significantly (Supplementary Figure 4, *P* < 0.05). These results indicated that knockdown of MIR210HG exerted tumor-suppressive effects in human breast cancer cell lines.

### MIR210HG regulates miR-1226-3p by acting as a ceRNA

**Figure 3:**
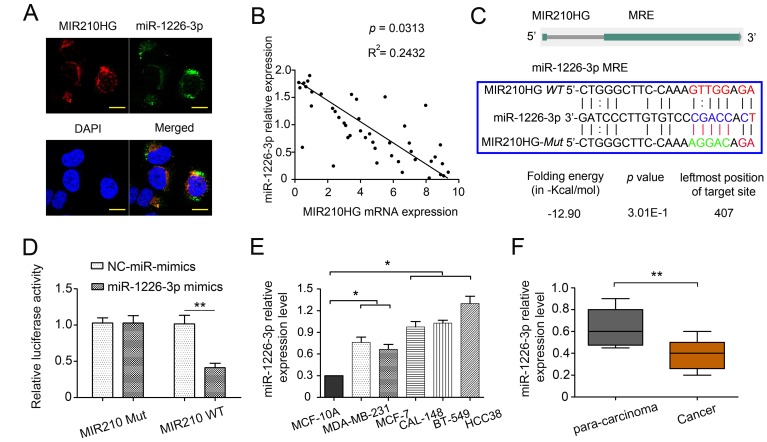
**MIR210HG regulates miR-1226-3p by acting as a ceRNA.** (**A**) MIR210HG colocalized with miR-1226-3p in IBC cell line MDA-MB-231 were detected by FISH assay, DAPI, 4′,6-diamidino-2-phenylindole, Magnification, × 400. (**B**) qRT-PCR results showed an inverse expression correlation between MIR210HG and miR-1226-3p in breast cancer tissues. (**C**) Diagram of wild-type (WT) and mutant (Mut) luciferase reporter plasmids, a putative miR-1226-3p target site in the 3′-UTR of MIR210HG mRNA was predicted in a bioinformatics analysis. (**D**) Luciferase reporter assay in human MDA-MB-231 cells co-transfected with WT and Mut type MIR210HG reporter and miR-1226-3p mimics. (**E**) qRT-PCR results showed that miR-1226-3p expression was lower in breast cancer cell lines (MDA-MB-231 and MCF-7 cells). (**F**) qRT-PCR results indicated miR-1226-3p was downregulated in IBC tissues compared with para-carcinoma groups. *p < 0.05, **p < 0.01.

To further explore the mechanisms underlying the oncogenic role of MIR210HG, the potential target miRNAs of MIR210HG were predicted using miRcode (http://mircode.org/), LncBase Predicted V.2 (http://carolina.imis.athena-innovation.gr/diana_tools/web/index.php?r=lncbasev2%2Findex-predicted) and starBase V3.0 (http://starbase.sysu.edu.cn/index.php). MiR-1226-3p and MIR210HG shared complementary sequences (Figure 3C). Thus, we focused on the interaction between MIR210HG and miR-1226-3p. FISH analysis showed that MIR210HG and miR-1226-3p colocalized in the cytoplasm in MDA-MB-231 (Figure 3A). This result suggested that MIR210HG may bind to miR-1226-3p in the cytoplasm. qRT-PCR results showed an inverse correlation between the expression levels of miR-1226-3p and MIR210HG in 45 paired IBC tissues (Figure 3B, *P* = 0.0313).

To ascertain whether MIR210HG can bind directly to miR-1226-3p at its miRNA response element (MRE), we constructed luciferase reporter plasmids, that contained wild-type (WT) or mutated (Mut) miR-1226-3p binding sites. MDA-MB-231 cells transfected with a miR-1226-3p mimic significantly diminished the luciferase activity of the MIR210HG-WT reporter vector, but not in the negative control or MIR210HG-Mut reporter vector transfected cells, validating the direct interaction between miR-1226-3p and MIR210HG (*P* < 0.01, Figure 3D). After overexpression of MIR210HG, the level of miR-1226-3p was significantly decreased (Supplementary Figure 4C–4D, *P* < 0.05). Our findings suggested that MIR210HG can inactivate miR-1226-3p via directly binding at the MRE. In addition, the expression of miR-1226-3p was determined in several human IBC cell lines and patients. In contrast to MIR210HG, miR-1226-3p expression was significantly lower in MDA-MB-231 and MCF-7 cells than that in CAL-148, BT-549 and HCC38 (Figure 3E, *P* < 0.05). Meanwhile, miR-1226-3p expression levels were significantly lower in IBC than the para-carcinoma tissues (Figure 3F, *P* < 0.01).

### MIR210HG regulates MUC1-C expression by competing for miR-1226-3p

**Figure 4:**
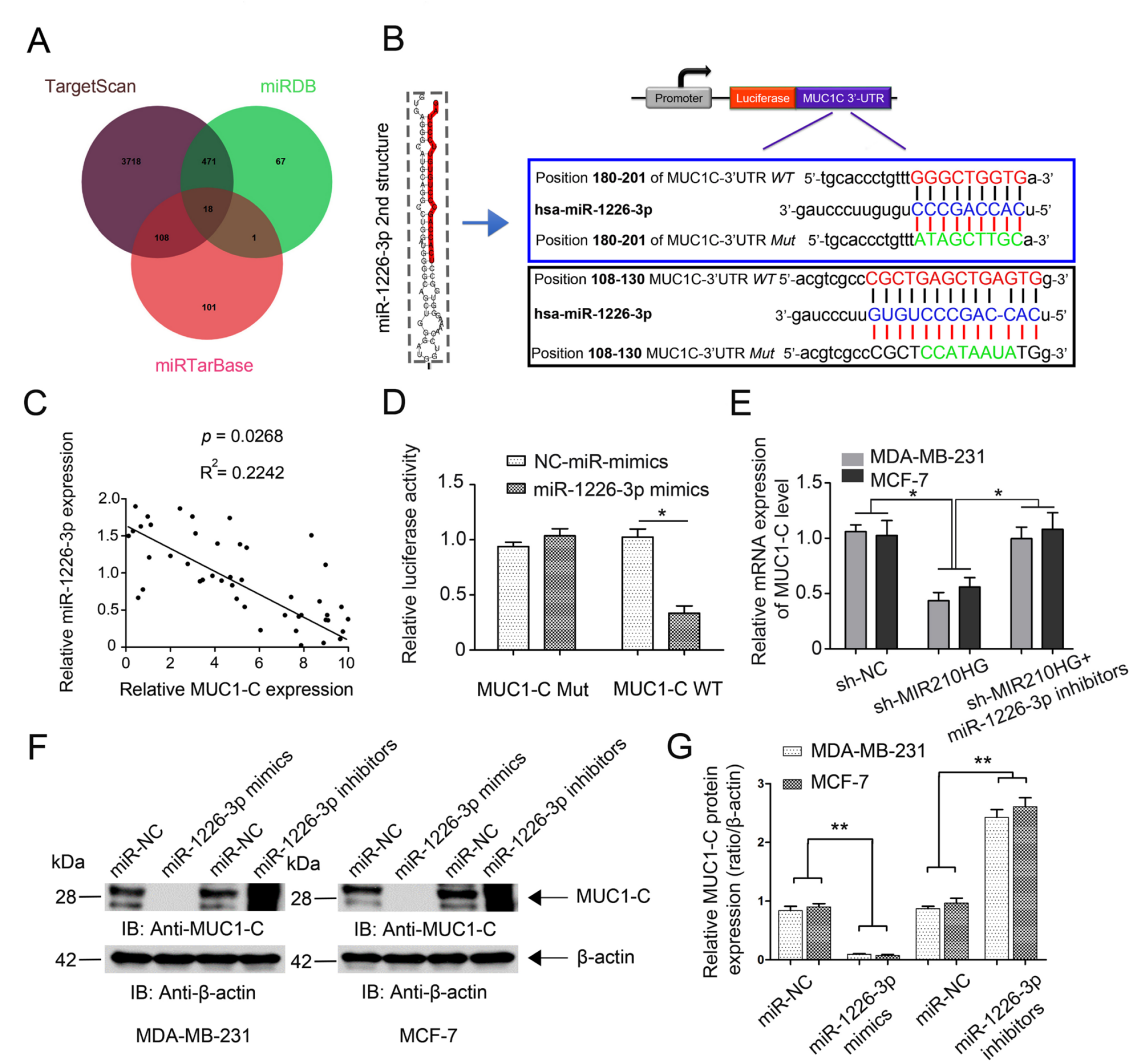
**MIR210HG regulates MUC1-C expression by competing for miR-1226-3p.** (**A**) Venn diagrams showing the number of potential miRNAs targeting the same genes (including MUC1-C gene), as predicted by three databases: TargetScan, miRDB and miRTarBase. (**B**) Ideograph of MUC1-C mRNA. The predicted miR-1226-3p binding site in the MUC1-C 3′-UTR. The sequence of wild-type (WT) and mutant (Mut) miR-1226-3p target sites in the MUC1-C 3′-UTR shown in frame. A point mutation was made in the seed region to block the binding between miR-1226-3p and mRNA, the sequence inside the blue frame is higher in the binding index. (**C**) qRT-PCR results showed a reverse correlation between MUC-1C mRNA and miR-1226-3p mRNA in IBC tissues. (**D**) Luciferase reporter assay showed that miR-1226-3p mimic transfection suppressed the luciferase activity of MUC-1C-WT reporter in MDA-MB-231 cells. (**E**) Rescue experiments confirm the mutual regulation of miR-1226-3p and MIR210HG. (**F**, **G**) miR-1226-3p mimics suppressed the expression of MUC-1C protein, whereas miR-1226-3p inhibitors reversed it in breast cancer cells. *p < 0.05.

To further investigate the mechanism of miR-1226-3p modulating tumor progression of IBC, we searched for potential downstream target genes of miR-1226-3p using three independent miRNA target-predicting algorithms (TargetScan, miRTarBase, and miRDB). We then concentrated on potential oncogenes associated with tumor metastasis out of 18 candidate target genes (Figure 4A). Next, bioinformatic prediction (TargetScan and miRDB) revealed a putative binding site for miR-1226-3p in the MUC1-C 3′- UTR with high complementarity (Figure 4B). We continued on to examine the correlation of MUC1-C with miR-1226-3p in 45 paired IBC tissues. Expectedly, the miR-1226-3p expression level was negatively associated with that of MUC1-C (Figure 4C, *P* = 0.0268). Similarly, we constructed luciferase reporter plasmids harboring wild type 3′-UTR of MUC1-C or the mutant on predicted binding site of miR-1226-3p. The results showed that miR-1226-3p mimic transfection suppressed the luciferase activity of MUC1-C-WT reporter in MDA-MB-231 cells (Figure 4D, *P* < 0.05), suggesting that MUC1-C was directly targeted by miR-1226-3p. The rescue experiment results showed that MIR210HG inhibition markedly suppressed the mRNA expression of MUC1-C, whereas miR-1226-3p inhibitors abrogated such a decrease in MUC1-C expression induced by MIR210HG suppression (Figure 4E, *P* < 0.05). Moreover, western blot analysis showed that miR-1226-3p mimics transfection suppressed the protein expression of MUC1-C, while miR-1226-3p inhibitors upregulated MUC1-C expression (Figure 4F–4G).

### MIR210HG promotes the growth of human breast tumor in murine xenograft models

**Figure 5:**
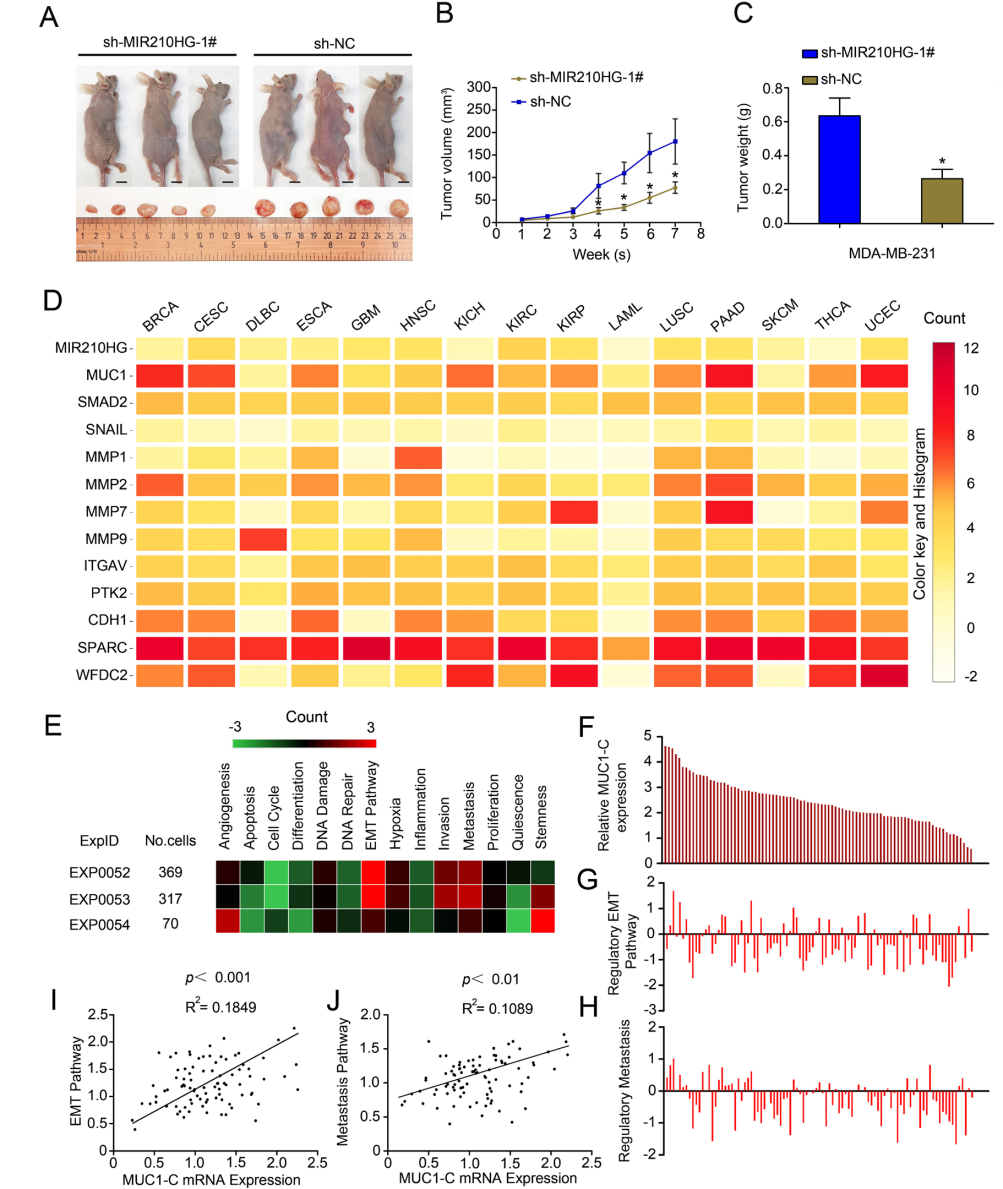
**MIR210HG promotes growth of breast tumor in murine xenograft models, and Single-cell sequencing results show that MIR210HG/MUC1 gene expression level is positively correlated with tumor metastasis pathway in invasive breast cancer.** (**A**) Effect of subcutaneously injection of MDA-MB-231 cells transfected with sh-NC or sh MIR210HG on the tumor growth. (**B** and **C**) Knockdown of MIR210HG expression significantly inhibited breast cancer cell growth in nude mice, and the tumor volume also weight were significantly reduced in the sh-MIR210HG group compared to that in the sh-NC group. (**D**) Mean-centered, hierarchical clustering of MIR210HG-related genes from TCGA multiple tumors data (UCSC Cancer Genomics Browser). Both MIR210HG and MUC1 are relatively high expression in a variety of solid tumors. (**E**) Correlations between the gene (MIR210HG and MUC1-C) of interest and functional states in 3 different single-cell datasets (CancerSEA). Single-cell datasets sequencing results show that MIR210HG and MUC1-C are closely related to EMT and tumor metastasis pathway. (**F**–**H**) Single-cell datasets of EXP0052 display 2 functional states that are significantly related to MUC1-C gene. (**I**) Functional relevance in different cell groups (EXP0052 single-cell datasets). Single-cell sequencing data show a significant positive correlation between MUC1-C gene expression and tumor EMT pathway regulation. (**J**) MUC1-C is positively associated with tumor metastasis similarly. *p < 0.05.

To study the biological role of MIR210HG in vivo, we established a xenograft tumor model in nude mice. MDA-MB-231 cells transfected with sh-MIR210HG or a control shRNA were subcutaneously injected into nude mice. Tumors derived from cells transfected with sh-MIR210HG were smaller in size and had a lower weight relative to the cells transfected with the control shRNA (*P* < 0.05, Figure 5A–5C). In order to determine the biological pathway that MIR210HG may be involved in, modules of multiple genes in PPI network were identified by the MCODE plugin in Cytoscape. Mutual exclusivity and co-occurrence network for miRNAs and circRNAs (associated with MUC1-C mRNA) with tumor suppressor genes and oncogenes were explored (Supplementary Figure 3A). PPI network of MUC1-C, the different protein sizes in the network imply the degree of specific gene in the PPI network (Supplementary Figure 3B). Mean-centered, hierarchical clustering of MIR210HG-related genes from TCGA multiple tumors data (UCSC Cancer Genomics Browser) had been detected in Figure 5D. Both MIR210HG and MUC1 show significant expression levels in a variety of solid tumors, including invasive breast cancer tissue. To further clarify the regulatory function of MIR210HG in breast tumor, we used ScRNA-seq database to analyze single cell sequencing data. Correlations between the genes of interest (MIR210HG and MUC1-C) and functional states in 3 different single-cell datasets (CancerSEA), EXP0052 [17], EXP0053 [18] and EXP0054 [19] were plotted. The results showed that MIR210HG and MUC1-C were closely related to EMT and tumor metastasis pathway (Figure 5E–5H). Functional relevance in EXP0052 single-cell datasets shown a significant positive correlation between MUC1-C gene expression and tumor EMT (Figure 5I, *P* < 0.001, R^2^ = 0.1849) and metastasis (Figure 5J, *P* < 0.01, R^2^ = 0.1089) pathway.

### MIR210HG/miR-1226-3p/MUC1-C axis in IBC metastasis-related EMT pathway

**Figure 6:**
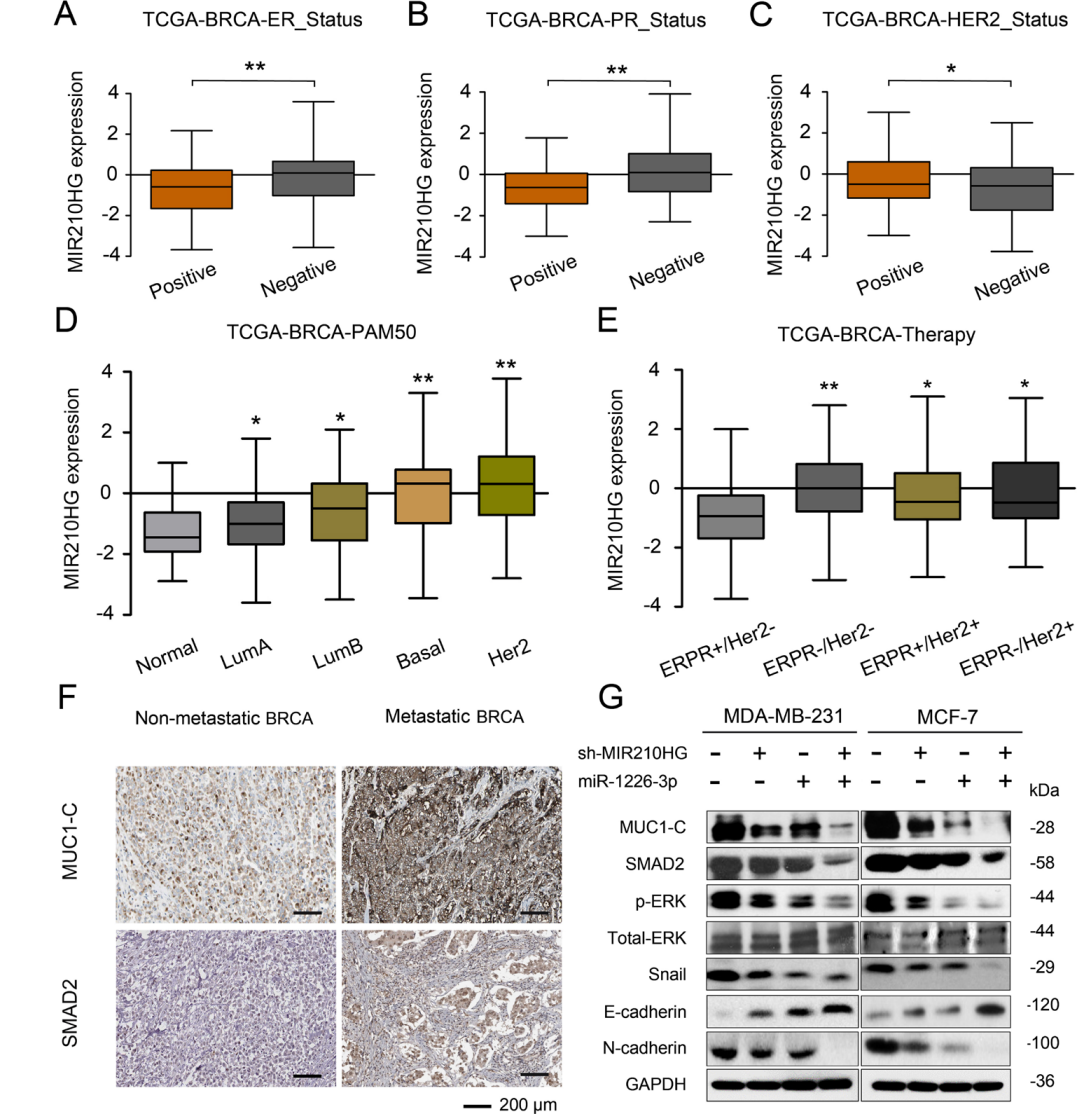
**MIR210HG is related to the molecular typing of malignant metastasis of IBC, and MIR210HG/miR-1226-3p/MUC1-C axis on tumor metastasis-related EMT pathway**. (**A**–**C**) Using the database of MD Anderson Cancer Center (TANRIC database), we found that MIR210HG was significantly associated with ER, PR and Her2 status in invasive breast cancer patients. (**D**) A 50-gene qPCR assay (PAM50) was developed to identify the intrinsic biological subtypes, the luminal A (LumA), luminal B (LumB), HER2-enriched (HER2-E), basal-like, and normal-like breast cancer subtypes. Analysis of sequencing data by TCGA-BRCA database showed that MIR210HG expression level was significantly correlated with PAM50 molecular typing. (**E**) TCGA-BRCA database sequencing data analysis showed that MIR210HG expression levels were associated with clinical treatment sensitivity in ERPR/Her2-guided typing. (**F**) MUC1-C was significantly overexpressed in IBC with lymph node metastasis compared to IBC non-metastasis (scale bars’ values are shown in each microphotograph, 50 μm). (**G**) WB analysis of MUC1-C, SMAD2, p-ERK/T-ERK, Snail, E-cadherin, and N-cadherin in MDA-MB-231 and MCF-7 cells. *p < 0.05, **p < 0.01.

**Figure 7:**
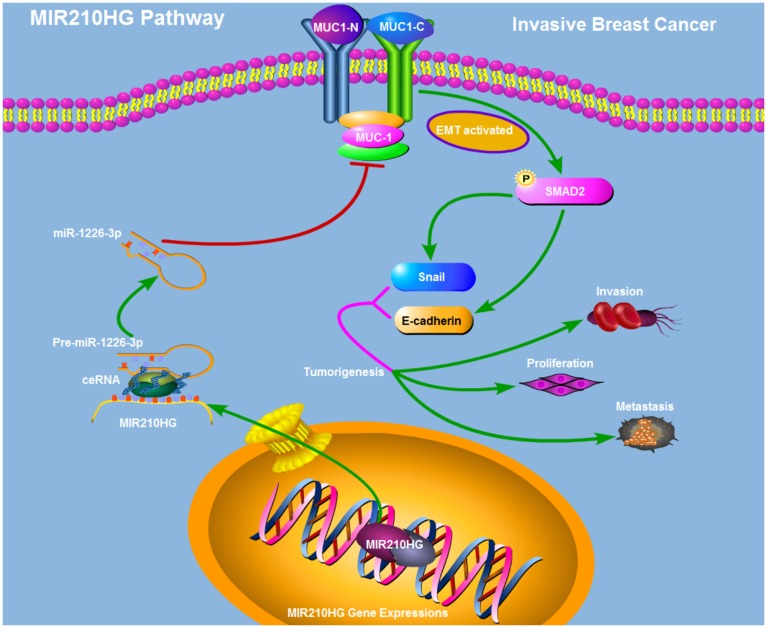
**Schematic model of the mechanism that MIR210HG promotes tumorigenesis and metastasis through a ceRNA pattern in IBC patients.** MIR210HG promotes MUC1-C/EMT pathway by competitively binding to miR-1226-3p.

To test the correlation between MIR210HG and clinical pathological parameters of breast cancer, taking advantage of TANRIC database, we found that MIR210HG was significantly associated with ER, PR and Her2 status in IBC patients (Figure 6A–6C). Thus further, in view of the advantages of a 50-gene qPCR assay (PAM50) for molecular typing of breast cancer, PAM50 has a high sensitive prognostic guidance for the clinical treatment of IBC. Analysis of sequencing data by TCGA-BRCA database showed that MIR210HG expression level was significantly correlated with PAM50 molecular typing (Figure 6D). More importantly, TCGA-BRCA database sequencing data analysis showed that MIR210HG expression levels were associated with clinical treatment sensitivity in ERPR/Her2-guided typing (Figure 6E). We used IHC to assess the protein levels of MUC1-C in metastatic breast cancer tissues. The results showed that MUC1-C was significantly higher in metastatic IBC than that in non-metastatic tumor, and a positive correlation was found between the expression levels of MUC1-C and SMAD2 protein (Figure 6F). Meanwhile, in vitro expression levels of key proteins in EMT pathway were also detected by western blotting (Figure 6G). Consistent with previous experiments, miR-1226-3p over-expression decreased the expression of MUC1-C, SMAD2, p-ERK, Snail, E-cadherin, while increased N-cadherin. This result can be synergistically enhanced by sh-MIR210HG. Taken together, sh-MIR210HG/miR-1226-3p demonstrated significant antitumoral activity both in *vitro* and in *vivo*. As summarized in Figure 7, the LncRNA MIR210HG in invasive breast cancer targets MUC1-C through ceRNA pattern and primarily regulates cell invasion, proliferation and metastasis.

## DISCUSSION

LncRNAs are a group of important players in various complex biological processes including normal development and disease pathogenesis [20–22]. To date, there have been few studies that have investigated the functions and mode(s) of action of MIR210HG in detail. Voellenkle et al. found that MIR210HG was induced by hypoxia in endothelial cells and mouse models of hindlimb ischemia, suggesting a regulatory function in hypoxia [23]. A coexpression network revealed a hub comprising lncRNAs (MIR210HG, SNHG1, and LOC729970) and mRNAs (RAB3D, DDX17, and SPNS2) that presumably mediate drug resistance in pancreatic cancer [24]. This information might illustrate its function in tumor biology [25]. However, the role and mechanism of MIR210HG in IBC has not been fully elucidated.

In this study, we first determined that MIR210HG is highly expressed in malignant breast tissues compared with adjacent normal tissues, and that the increased expression had a positive correlation with the tumor TNM stage. Previous reports have shown that MIR210HG is a ceRNA sponge for miRNAs, thereby de-repressing the target genes of these miRNAs in glioma and colorectal adenocarcinoma. Therefore, we hypothesized that MIR210HG acts as a ceRNA in IBC. Previous studies have shown that, compared with healthy controls, the expression of miR-1226-3p was significantly higher in stomach tumors but lower in colorectal tumors [26]. We further demonstrated that miR-1226-3p expression levels were noticeably downregulated in IBC tissues and cells. Furthermore, miR-1226-3p expression levels were negatively correlated with those of MIR210HG in IBC tissues. Bioinformatic analysis showed that MIR210HG possesses one conserved target site for miR-1226-3p binding. Further, FISH results showed intracellular colocalization of MIR210HG and miR-1226-3p, both of which were mainly concentrated in the cytoplasm. A dual-luciferase reporter assay confirmed that miR-1226-3p reduced the luciferase activity of MIR210HG. In addition, MIR210HG inhibition markedly suppressed IBC proliferation and invasiveness process. In our study, miR-1226-3p overexpression decreased MUC1-C expression, which could be upregulated by miR-1226-3p inhibitors. Also, miR-1226-3p notably decreased the luciferase activity of MUC1-C. These results indicated that MUC1-C was a direct target of miR-1226-3p. Taken together, our findings indicate that MIR210HG promotes IBC by negatively regulating the miR-1226-3p/MUC1-C axis.

Mucin 1 (MUC1) is a heterodimeric protein that is aberrantly overexpressed by diverse types of carcinomas, including those of the breast and lung [27–29]. The National Cancer Institute Project for the Acceleration of Translational Research ranked MUC1 as the second-most promising cancer antigen among 75 candidates [30, 31]. MUC1 is translated as a single polypeptide that autocleaves into two subunits [32–34]. MUC1-N forms a complex with the MUC1 C-terminal (MUC1-C) subunit at the cell membrane and is shed from the surface of cancer cells in association with increased plasma levels [35, 36]. Recent research shows that MUC1-C induces EMT by stimulating the NF-κB p65 pathway and, consequently, the EMT-related transcription factor ZEB1. MUC1-C drives a program inducing EMT and stem cell phenotypes, epigenetic reprogramming and immune evasion. Our experiments also altered the expression of MIR210HG, which in turn affects the expression of MUC1 protein, and ultimately regulates the EMT process associated with tumor metastasis in breast cancer. By contrast, the nonshed MUC1-C subunit, which functions as an oncoprotein, represents an attractive target for the development of mAb-based therapeutics [37–39]. In concert with these findings, overexpression of MUC1-C in breast and lung carcinomas is associated with induction of gene signatures that are predictive of significant decreases in disease-free and overall survival, further supporting the attractiveness of MUC1-C as a cancer target [40, 41].

## MATERIALS AND METHODS

### Patients and samples

45 pairs of IBC tissue samples and neighboring non-cancerous tissues (collected postoperatively from January 2012 to December 2013) used in this study were obtained from the Department of General Surgery, Shanghai Tenth’s People Hospital, Shanghai Putuo People’s Hospital of Tongji University and Department of Pathology, Beijing Chao-Yang Hospital of Capital Medical University. These patients did not receive any radiotherapy and chemotherapy before surgery. Tumors were classified according to the tumor-node-metastasis (TNM) system of classification (2014 version). The ethical approval of this study protocol was authorized from the Ethics Committee of Shanghai Tenth’s People Hospital and Shanghai Putuo People’s Hospital of Tongji University, and detailed written consent was obtained from all enrolled subjects. The general clinical and laboratory features of the IBC patients were summarized in Table 1.

**Table 1:** Association between MIR210HG and clinicopathological characteristics in invasive breast cancer (IBC).

**Characteristics**	**No.(n=45)**	**MIR210HG**		***p* value**
		**High (%)**	**Low (%)**	
Age (years)				0.361
≤55	29	13(28.0%)	16(35.5%)	
>55	16	7(15.5%)	9(20.0%)	
Tumor size				<0.001*
≤1.5 cm	14	3(6.7%)	11(24.4%)	
>1.5 cm	31	14(31.1%)	17(37.8%)	
Climacteric syndrome				0.473
yes	21	6(13.3%)	15(33.3%)	
no	24	11(24.4%)	13(29%)	
Lymph node				0.003*
Yes	17	7(15.6%)	10(22.2%)	
no	28	9(20%)	19(42.2%)	
TNM stage				<0.001*
I-II	35	12(26.7%)	23(51.1%)	
III-IV	10	4(8.9%)	6(13.3%)	
Histological grade				0.832^#^
G1	4	0(0%)	4(8.9%)	
G2	23	7(15.6%)	16(35.5%)	
G3	18	5(11.1%)	13(28.9%)	

^#^ Fisher’s exact test, *p < 0.05, statistically significant.

### Cell culture

The human IBC cell lines (MDA-MB-231 and MCF-7) were obtained from ATCC (American Type Culture Collection, USA) and cultured in a humidified incubator at 37°C with an atmosphere of 5% CO_2_. MDA-MB-231 and MCF-7 cells were maintained in Leibovitz’s L-15 Medium, Eagle’s Minimum Essential Medium (EMEM), respectively, containing 10% fetal bovine serum (Thermo Fisher Scientific, USA) and Penicillin (100 U/ml)-Streptomycin (0.1 mg/ml) Solution.

### RNA extraction and qRT-PCR assay

Total RNA was extracted with TRIzol (Invitrogen, USA) and reversely transcribed into cDNA following the manufacturer’s protocol. Then, qRT-PCR was performed with Power SYBR Green (Takara, Japan). Data were normalized to GAPDH expression. The expression levels of miR-1226-3p were determined using a TaqMan Human MiRNA Assay Kit (Invitrogen, USA) with normalization to U6. PCR primers were listed in Table 2, and the relative expression levels were calculated using the 2^−ΔΔCt^ method.

**Table 2:** List of primers and target sequence used in this paper.

**Gene**	**Primer**	**Sequence(5′-3′)**
MUC-1C	forward	CCTACCATCCTATGAGCGAGTAC
	reverse	GCTGGGTTTGTGTAAGAGAGGC
MIR210HG	forward	GCTTGGTAGAGTGTCACGCC
	reverse	CATCTGACCGAGCCAGTTTG
miR-1226-3p	forward	CCCCTGCGTGTTTTATGAAG
	reverse	CCTGTACTGGGGAAGTTCA
MIR210HG^*^	sh-miR210HG-1^*^	GGAGGGAATTAGAAGCGTT
	sh-miR210HG-2^*^	GCATTTACAGGCCAGCCTA
U6	forward	GCTTCGGCAGCACATATACTAAAAT
	reverse	CGCTTCACGAATTTGCGTGTCAT
GAPDH	forward	CCATGTTCGTCATGGGTGTG
	reverse	GGTGCTAAGCAGTTGGTGGTG

^*^ The target sequence of sh-RNAs.

### Plasmid constructs and transfection

MIR210HG cDNA was cloned into the mammalian expression vector pLenti-GIIICMV-Puro vector from Genepharm (Shanghai, China). The viral supernatants were added to MDA-MB-231 cells to establish stable MIR210HG overexpressing cell lines. Cells were further subjected to puromycin (2 μg/ml) selection for one week. The miR-1226-3p inhibitor and mimic were purchased from GenePharma. To construct luciferase reporter vectors, MUC1-C 3′-untranslated regions (UTR) and MIR210HG cDNA fragment containing the predicted potential miR-1226-3p binding sites or mutant sites were amplified by PCR. Cells were plated in 6-well plates and transfected using lipofectamine-3000 (Invitrogen, USA) according to manufacturer’s instructions. Cells were collected for real-time PCR or western blot 48 h after transfection. The target sequence of sh-RNAs were described in Table 2.

### Microarray analysis and TCGA dataset analysis

Microarray analysis for the expression of noncoding RNAs and TCGA data set analysis was performed by Department of Data Sciences, Dana-Farber Cancer Institute of Harvard Medical School (Boston, USA). The microarray data used in this paper were downloaded from the Gene Expression Omnibus database GEO (https://www.ncbi.nlm.nih.gov/geo/), and the accession numbers were GSE80038 and GSE113851 [42, 43]. The preprocessed level-3 RNA-seq data and the corresponding clinical information of tumor patients were collected from the Cancer Genome Atlas (TCGA) database (https://cancergenome.nih.gov/). We used the edgeR package to perform the differential analysis, classify and draw tumor-gene differentially expressed box plot, (http://www.bioconductor.org/packages/release/bioc/html/edgeR.html) and used the pheatmap package to perform the cluster analysis (https://cran.r-project.org/web/packages/pheatmap/index.html). Sva R package was used to remove the batch effect. Noncoding RNAs/genes with adjusted p < 0.05 and absolute fold changes (FC) > 1.5 were considered differentially expressed. We used a Pearson χ^2^ test to examine the association of noncoding RNAs with genes. Querying which functional states the gene (including MIR210HG and MUC1) is related to across breast cancer stages, and many bulk RNA-seq datasets to discover differentially expressed genes or identify cell types in breast tumor tissues, we used ScRNA-seq database (http://biocc.hrbmu.edu.cn/CancerSEA/home.jsp) to classify the function of the target gene from single-cell sequencing data.

### Proliferation and colony formation assay

Proliferation of cells transfected with indicated vector was measured by MTT assay kit (detected at 24, 48, 72, 96 and 120 h, respectively). We seeded cells into 96-well plates (1 × 10^3^ cells per well). After indicated culture time, medium was removed. MTT reagent was added to each well. After 3 hr incubation at 37 °C, MTT solvent was added to each well. The absorbance was measured at 520 nm using a microplate reader. For colony formation experiments, cells were resuspended at 1 × 10^3^ cells/well and cultured for about 4 days until macroscopic colonies appeared. Finally, the colonies were stained with crystal violet (Beyotime, China) for 30 minutes.

### Transwell invasion assay

Transwell assay was performed to measure invasion of breast cancer cells using Matrigel Chambers (8-mm pores; BD Biosciences, USA). Briefly, 10^4^ cells (in 150 μL) were plated in the upper chambers with serum-free EMEM and EMEM supplemented with 10% FBS in the lower chamber. After incubation at 37°C for 12 hours, cells were fixed with ice-cold methanol for 30 min and stained with 0.1% crystal violet for 5 min. Invading cells on the lower chamber were calculated using photographic images.

### Luciferase reporter assay

70-80% confluent Endothelial Progenitor Cells in 24-well plates were cotransfected with indicated luciferase reporter vector (200 ng) and appropriate miRNA (50 nM, mimics and Inhibitors) using lipofectamine-3000 reagent (Invitrogen, USA). 72 hours after transfection, cells were washed and lysed with passive lysis buffer (Sigma, USA). Luciferase activity was analyzed using a dual luciferase assay (Aibosi). Relative luciferase activity was obtained by normalization to Renilla luciferase activity.

### Western blots

Proteins were extracted by RIPA buffer. After determination of protein concentrations, 45 μg of total protein per lane were separated by SDS-PAGE and transferred onto PVDF membranes (Millipore, USA). The membranes were blocked with 5% fat-free milk in TBS-T buffer and then incubated with antibodies against MUC1-C (CT2 antibody, Abcam, USA) at 4°C overnight. After washing, a horseradish peroxidase-conjugated secondary antibody was used for protein detection. β-actin (Abcam, USA) was used as an internal control.

### Fluorescence in situ hybridization (FISH) assay

FISH assay was performed to detect subcellular location of MIR210HG and miR-1226-3p. Cells were grown on cover slips for 48 hours and fixed. The slides were deparaffinized with xylene and rehydrated with series percentage of alcohol, followed by incubation with 4% paraformaldehyde for 30 min. Then the slides were washed and incubated with prehybridization solution for 1 hour at 37°C. Digoxin-labeled MIR210HG and miR-1226-3p probes were denatured at 85°C for 5 min and hybridized for 24 hours at 37°C. Subsequently, after blocking, the sections were incubated with HRP conjugated anti-digoxigenin at 37°C for 1 h. After washing with PBS, TSA-488 solution (Invitrogen, USA) was added to the slides. The sections were counterstained with 4, 6-diamidino-2-phenyl-indole (DAPI) for cell nucleus.

### Animal experimental model

Female BALB/c athymic nude mice (4-5 weeks) were purchased form the Shanghai Experimental Animal Center of Chinese Academic of Sciences (Shanghai, China). Two groups (5 mice in each group) were kept under pathogen-free conditions. For *in vivo* metastasis experiments, sh-MIR210HG or sh-MIR210HG-control expressing cells (1.5 × 10^6^ cells/mouse) were subcutaneously inoculated under the right lower limbs of nude mice. Tumors were measured twice weekly with digital calipers. Tumor volume was calculated using the equation: tumor volume (mm^3^) = L(length) × W^2^ (width) × 0.5. Mice were euthanized by ether inhalation at the end of the study. Tumors were excised and fixed in formalin for other experiments.

### Immunohistochemistry

Detection of MUC1-C and SMAD2 were performed on 5-μm thick paraffin sections with the indicated antibodies. Briefly, the sections were incubated with primary antibodies (Abcam, ab109185, dilution 1:500) overnight at 4°C, followed by incubation with an secondary antibody (dilution 1:200, Cell Signaling Technology, Danvers, USA) at 37°C for 30 min.

### Statistical analysis

All in vitro experiments were done in triplicate. The results were presented as the mean ± SD(standard deviation). Statistical comparisons were performed using one-way ANOVA or paired t-test. The association of MIR210HG expression with clinicopathological parameters was evaluated by Fisher’s exact test. Spearman Pearson correlation analysis was conducted to assess the correlation between MIR210HG and miR-1226-3p expression. All human data were analyzed using SPSS 19.0 (SPSS Inc., Chicago, IL, USA) and *P* value < 0.05 as statistically significant.

### Ethics approval

All procedures performed in studies involving human participants were in accordance with the ethical standards of the institutional and/or national research committee and with the 1964 Helsinki declaration and its later amendments or comparable ethical standards. And the present study was approved by the Ethics Committee of Shanghai Putuo People’s Hospital of Tongji University. All procedures performed in studies involving animals were in accordance with the ethical standards of the institution or practice at which the studies were conducted.

## CONCLUSIONS

Taken together, our study revealed the oncogenic functions of MIR210HG in IBC. Elevated levels of MIR210HG are positively correlated with tumor progression. MIR210HG functions as a ceRNA for miR-1226-3p to promote IBC progression partially through mucin-1c signaling. Further, our research provides further insight into the molecular mechanism of MIR210HG in IBC tumorigenesis. This understanding may aid in the advancement of lncRNA-directed diagnosis and therapy for IBC.

## Supplementary Material

Supplementary Figures
