# Supplementary material for: The long noncoding RNA MIR210HG promotes tumor metastasis by acting as a ceRNA of miR-1226-3p to regulate mucin-1c expression in invasive breast cancer
Source: Aging (Albany NY). 2019 Aug 10;11(15):5646–65. doi: 10.18632/aging.102149 (PMC6710038; doi:10.18632/aging.102149)
Supplement: Supplementary Figures [file aging-11-102149-s001.pdf]

SUPPLEMENTARY FIGURES

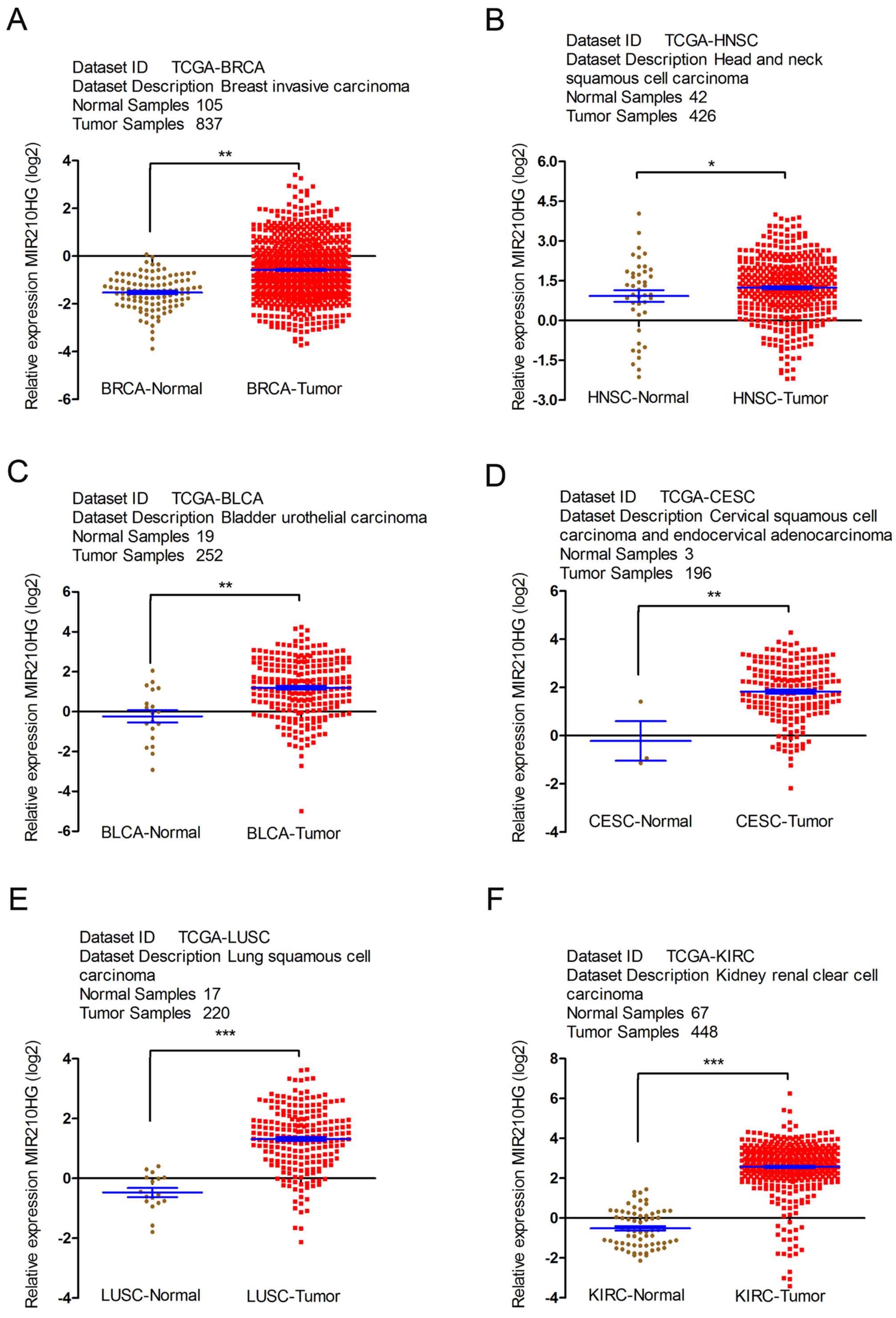

Supplementary Figure 1. MIR210HG expression in TCGA pan-cancer data set and normal samples. (**A**) Breast invasive carcinoma, BRCA; (**B**) Head and Neck squamous cell carcinoma, HNSC; (**C**) Bladder Urothelial Carcinoma, BLCA; (**D**) Cervical squamous cell carcinoma and endocervical adenocarcinoma, CESC; (**E**) Lung squamous cell carcinoma, LUSC; (**F**) Kidney renal clear cell carcinoma, KIRC. *p <0.05, **p < 0.01 and ***p < 0.001 versus corresponding normal.

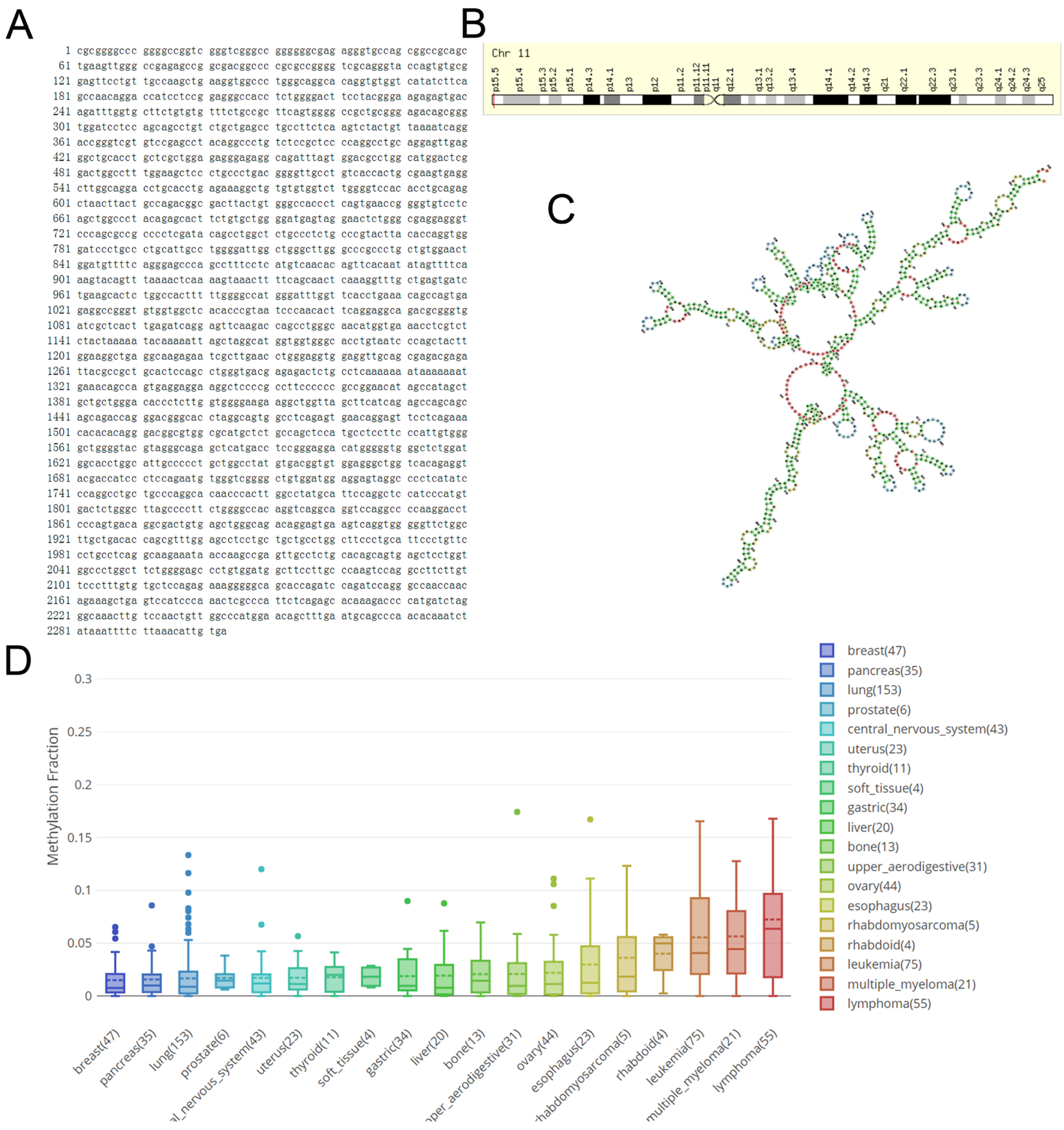

Supplementary Figure 2. (**A**) The full length sequence of MIR210HG from the GeneCards website (https://www.genecards.org/). (**B**) MIR210HG Gene in genomic location: 11p15.5, bands according to Ensembl, locations according to GeneLoc. (**C**) Secondary structure of MIR210HG from GeneCards website. (**D**) Methylation index of MIR210HG in different tissues of human body, the methylation index is low in breast tissues specifically, suggesting the possible mechanism of high expression level of MIR210HG in breast tissues.

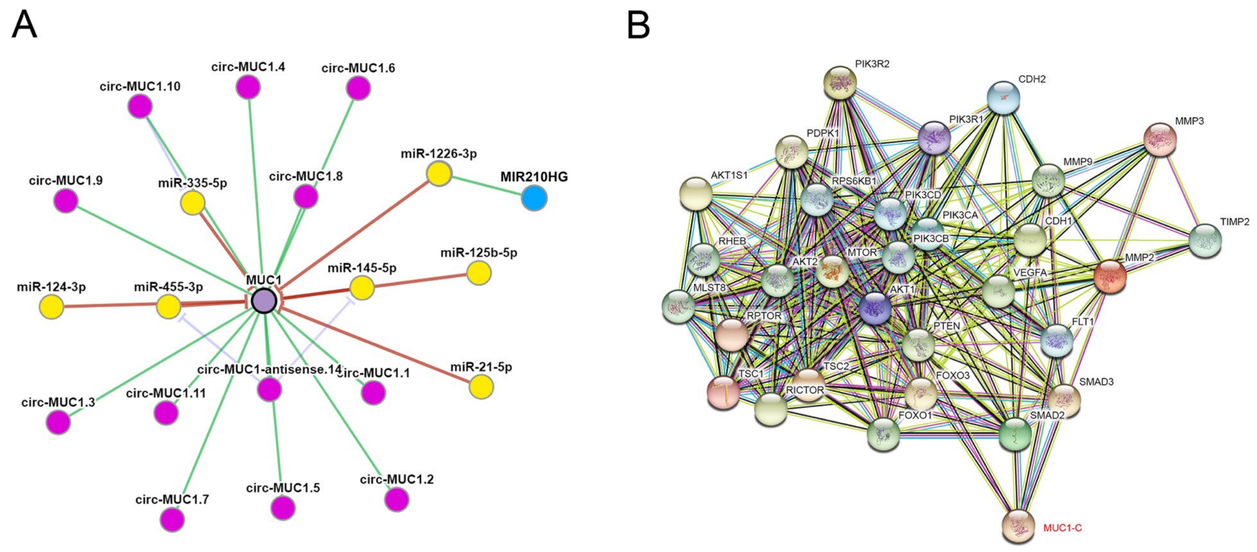

Supplementary Figure 3. (**A**) Mutual exclusivity and co-occurrence network for miRNAs and circRNAs (associated with MUC1-C mRNA) with tumor suppressor genes and oncogenes. (**B**) PPI network of MUC1-C, the different protein sizes in the network imply the degree of specific gene in the PPI network. Abbreviation: PPI, protein-protein interactions.

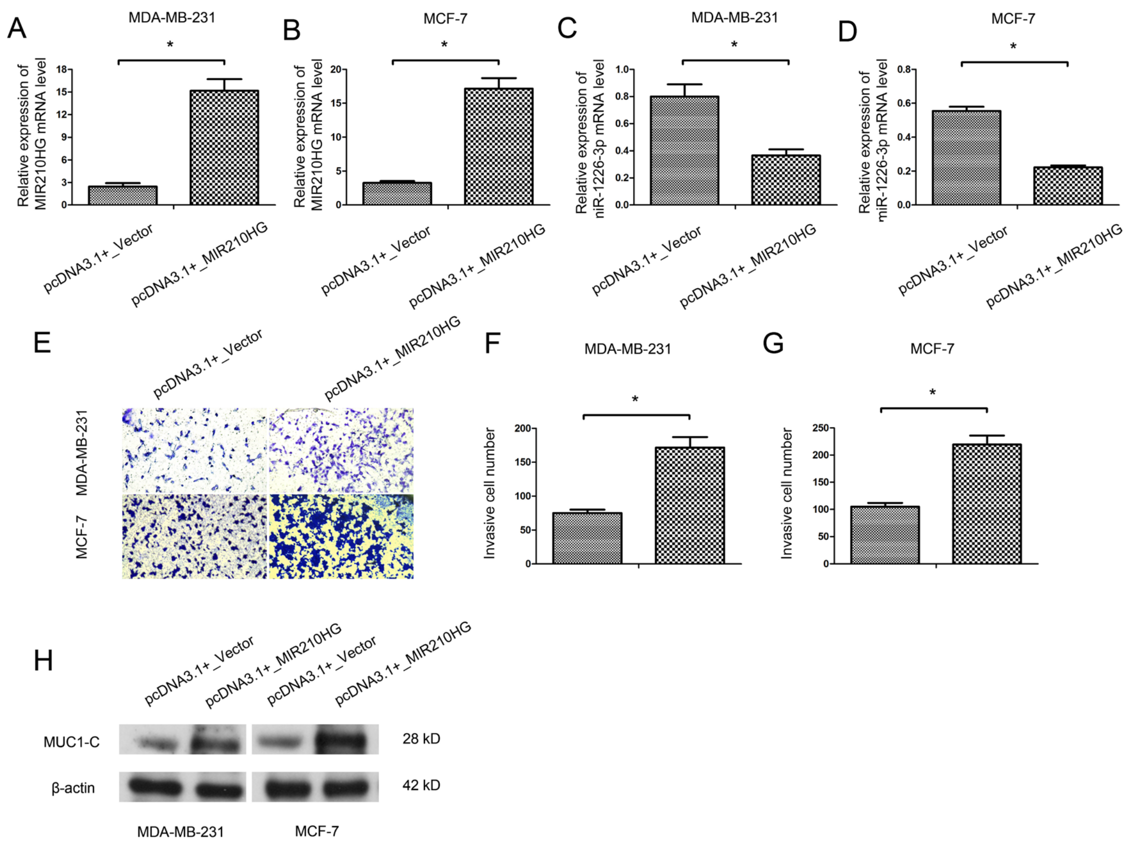

Supplementary Figure 4. MIR210HG overexpression promotes breast cancer cells invasion in vitro. Relative expression of MIR210HG (**A**, **B**) and miR-1226-3p (**C**, **D**) in pcDNA3.1^{+}_MIR210HG transfected compared with pcDNA3.1^{+}_vector transfected MDA-MB-231 and MCF-7 cells. (**E**–**G**) Transwell assays were performed to evaluate the invasion of MIR210HG overexpressed compared with NC MDA-MB-231 and MCF-7 cells. (**H**) Western blot analysis of MUC1-C upon MIR210HG overexpression. β-actin was used as an internal control. *$p < 0.05$.
